# Functional imaging of the exposed brain

**DOI:** 10.3389/fnins.2023.1087912

**Published:** 2023-02-09

**Authors:** Sadaf Soloukey, Arnaud J. P. E. Vincent, Marion Smits, Chris I. De Zeeuw, Sebastiaan K. E. Koekkoek, Clemens M. F. Dirven, Pieter Kruizinga

**Affiliations:** ^1^Department of Neuroscience, Erasmus MC, Rotterdam, Netherlands; ^2^Department of Neurosurgery, Erasmus MC, Rotterdam, Netherlands; ^3^Department of Radiology and Nuclear Medicine, Erasmus MC, Rotterdam, Netherlands; ^4^Netherlands Institute for Neuroscience, Royal Dutch Academy for Arts and Sciences, Amsterdam, Netherlands

**Keywords:** functional imaging, brain mapping, craniotomy, neuronal activity, surgical decision-making, imaging techniques, exposed brain, functional ultrasound

## Abstract

When the brain is exposed, such as after a craniotomy in neurosurgical procedures, we are provided with the unique opportunity for real-time imaging of brain functionality. Real-time functional maps of the exposed brain are vital to ensuring safe and effective navigation during these neurosurgical procedures. However, current neurosurgical practice has yet to fully harness this potential as it pre-dominantly relies on inherently limited techniques such as electrical stimulation to provide functional feedback to guide surgical decision-making. A wealth of especially experimental imaging techniques show unique potential to improve intra-operative decision-making and neurosurgical safety, and as an added bonus, improve our fundamental neuroscientific understanding of human brain function. In this review we compare and contrast close to twenty candidate imaging techniques based on their underlying biological substrate, technical characteristics and ability to meet clinical constraints such as compatibility with surgical workflow. Our review gives insight into the interplay between technical parameters such sampling method, data rate and a technique’s real-time imaging potential in the operating room. By the end of the review, the reader will understand why new, real-time volumetric imaging techniques such as functional Ultrasound (fUS) and functional Photoacoustic Computed Tomography (fPACT) hold great clinical potential for procedures in especially highly eloquent areas, despite the higher data rates involved. Finally, we will highlight the neuroscientific perspective on the exposed brain. While different neurosurgical procedures ask for different functional maps to navigate surgical territories, neuroscience potentially benefits from all these maps. In the surgical context we can uniquely combine healthy volunteer studies, lesion studies and even reversible lesion studies in in the same individual. Ultimately, individual cases will build a greater understanding of human brain function in general, which in turn will improve neurosurgeons’ future navigational efforts.

## 1. Introduction

Neurosurgical practice provides rare and direct access to the exposed human brain, which is otherwise safely hidden under the skull and dura. This exposure provides a unique opportunity for functional imaging of the brain, especially when harnessing techniques which have limited transcranial penetrability. Imaging the exposed brain can have multiple, synergistic purposes: (1) ensuring that neurosurgical procedures such as tumor removals or electrode placements are effective and safe by mapping out brain function, and as an added benefit (2) increasing our neuroscientific understanding of brain function in general.

Currently, Electrocortical Stimulation Mapping (ESM)—which dates back to the early years of the 20th century (Pendleton et al., 2012)—is considered to be the neurosurgeon’s most direct tool for intra-operative, functional feedback. During awake craniotomy for tumor removal, ESM is used to interact with the brain by stimulating regions of interest of the cortex to create functional maps around the tumor which help guide the neurosurgeon’s intra-operative decision-making. In the context of neuro-oncology, patient outcomes in terms of survival (Li et al., 2015; Brown et al., 2016; Wijnenga et al., 2018; Gerritsen et al., 2019a), post-operative neurological functioning (Gerritsen et al., 2019a) and quality of life (Drewes et al., 2018) are a direct result of this decision-making between removing as much of the tumor as possible while minimizing the damage to surrounding functional brain areas, also known as reaching “maximum safe tumor resection” (Li et al., 2015). Similarly, in epilepsy surgery, accurate delineation of the epileptogenic zone is essential for radical resection of the pathological tissue, while ensuring surgical safety in terms of post-operative neurological deficits (Rosenow and Lüders, 2001; Vakharia et al., 2018). ESM has proven to improve surgical outcome and safety (Gerritsen et al., 2019a,b) and holds a unique position in the landscape of functional brain mapping as it depends on direct disruption or production of functional behavior, rather than correlation-based mapping as many imaging-based techniques use. Nevertheless, ESM remains a non-standardized technique with low spatial resolution (∼1 cm), limited penetration depths (<1 cm) and side-effects such as seizure elicitation (Ritaccio et al., 2018).

In this review, we will compare and contrast current and emerging techniques which could be used for real-time, intra-operative functional brain mapping. We will focus specifically on those techniques which especially benefit from the fact that the brain is exposed: in other words, those techniques which have limited transcranial penetrability. As there are no gold standard real-time imaging techniques as of yet, we will consider all potential techniques, regardless of whether they are experimental or clinical, applied in humans or in animals.

We will initially categorize these techniques based on the biological substrate underlying their functional imaging, which will serve as the framework for discussing each technique’s technical characteristics such as the sampling methods, spatiotemporal resolution and penetration depth.

### 1.1. Defining functionality

We will be discussing so-called “functional imaging techniques,” which we define as any technique which has the ability to visualize functionality of brain tissue in a non-invasive manner. We will define “functionality” of brain tissue as the tissue’s ability to produce specific neuronal activity, functional tasks or states, such as moving the hand or repeating a word. In the neurosurgical context, we assume that brain tissue functionality is at least present in so-called eloquent brain areas: areas of the brain “*that speak to readily identifiable neurologic function and, if injured, result in a disabling neurologic deficit”*(Spetzler and Martin, 1986; Kahn et al., 2017). This in contrast to pathological tissue such as tumor tissue, epileptic, tumor-invaded, necrotic or damaged brain tissue which displays no such functionality. Other key terminology used throughout this paper is summarized in the glossary in Appendix A.

## 2. Biological substrates underlying functionality

**FIGURE 1:**
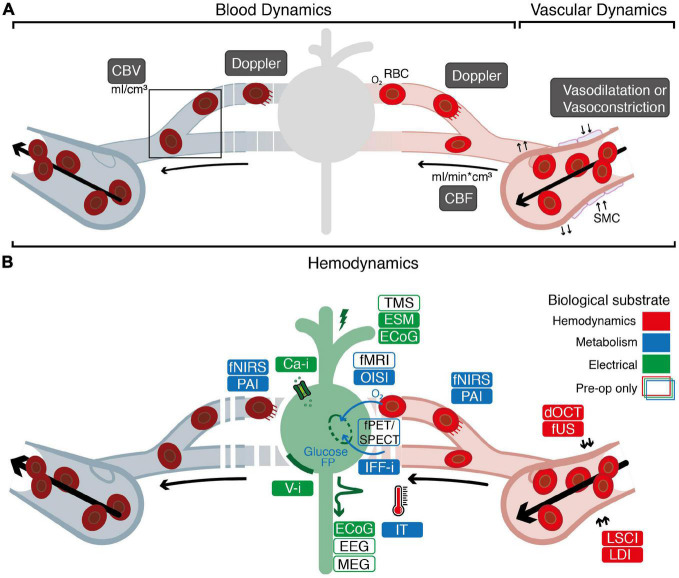
Overview of the biological substrates underlying different functional imaging techniques. **(A)** Graphical overview of the relationship between blood dynamics, vascular dynamics and hemodynamics. Blood dynamics consists of changes in CBV (the volume of blood in a volume of brain) and changes in CBF (the speed with which a volume of blood perfuses a volume of brain). Vascular dynamics specifically encompasses vasoconstriction and vasodilatation, mediated by smooth muscle cells (SMCs) surrounding larger veins, arteries and smaller arterioles. “Hemodynamics” is used as an overarching term encompassing blood and vascular dynamics. **(B)** Electrical techniques (*depicted in green*) rely on detection of electrical signal (ECoG, EEG, MEG) or direct proxies thereof (Ca-I, V-i) to measure neuronal activity. Alternatively, techniques such as ESM or ECoG rely on introduction of electrical current into the brain to actively produce or interrupt neuronal activity. Through the principles of neurovascular coupling (NVC), electrical neuronal activity is linked to changes in hemodynamics, which underlies techniques such as dOCT, fUS, LDI, and LSCI (*depicted in red*). This neurovascular response is closely interwoven with the metabolic requirements for neuronal activity known as neurometabolic coupling (NMC). Techniques such as IFF-I, IT, OISI, PAI, fNIRS, fPET/SPECT, and fMRI rely primarily on measurements of metabolites as a proxy for functional activity (*depicted in blue*). White boxes indicate pre-operative techniques specifically. ECoG, electrocorticography; EEG, electroencephalography; MEG, magnetoencephalography; ESM, electrocortical stimulation; TMS, transcranial magnetic stimulation; Ca-i, calcium-imaging; V-i, voltage-imaging; NVC, neurovascular coupling; PAI, photo-acoustic imaging; fNIRS, functional near-infrared spectroscopy; dOCT, Doppler optical coherence tomography; fUS, functional ultrasound; LDI, laser Doppler imaging; LSCI, laser speckle contrast imaging; RBC, red blood cells; NMC, neurometabolic coupling; IFF-I, intrinsic functional fluorescence-imaging; IT, infrared thermography; OISI, optical intrinsic signal imaging; fMRI, functional magnetic resonance Imaging; fPET, functional position emission tomography; SPECT, single-photon emission computed tomography; FP, flavoprotein.

On a single neuron level, functional activity consists of action potentials which propagate along axons, resulting in neuronal activation on a time-scale of just several milliseconds (Barnett and Larkman, 2007). Through the principles of Neurovascular Coupling (NVC) and Neurometabolic Coupling (NMC), electrical neuronal activity is linked to changes in hemodynamics as well as consumption of metabolites such as oxygen and glucose (Iadecola, 2017). These processes are complex and widely studied (Iadecola, 2017), yet their exact physiology remains to be elucidated. With an increase in activation of neurons, energy consumption increases, which needs to be metabolized from glucose and oxygen supplied by red blood cells (RBCs) through local capillaries (Hyder, 2010; Petzold and Murthy, 2011; Huneau et al., 2015; Iadecola, 2017). Initially, a depletion of the local oxygen supply is observed (Ances, 2004; Iadecola, 2004). Consequently, this depletion is met with a 1–2 s delayed hemodynamic response, which involves local and upstream vessel dilatation and increase in Cerebral Blood Flow (CBF) and Cerebral Blood Volume (CBV), known as “functional hyperemia” (Ances, 2004; Iadecola, 2004). These changes in blood and vascular dynamics (collectively known as hemodynamics) (Figure 1A)–and specifically the movement of RBCs—may induce a Doppler-effect (Uppal and Mogra, 2010) when interrogated with hemodynamics-based imaging techniques. Techniques that rely on NVC or NMC are considered a *proxy* of neuronal activity (Figure 1B). The consequences of this for accuracy of functional images seems highly dependent on the contextual factors such as the functional task (Gould et al., 2003), the region of imaging (Ances et al., 2008), the use of anesthesia (Aksenov et al., 2015) or even the patient’s specific underlying pathology (Girouard and Iadecola, 2006; Pak et al., 2017). In peri-tumoral tissue, for example, vascular changes can lead to neurovascular uncoupling and hence false negative responses (Pak et al., 2017). For more background on the mechanisms underlying NVC and NMC we refer to the following references (Hillman, 2014; Phillips et al., 2015; Iadecola, 2017; Drew, 2019; Kaplan et al., 2020).

## 3. Visualizing functionality

**FIGURE 2:**
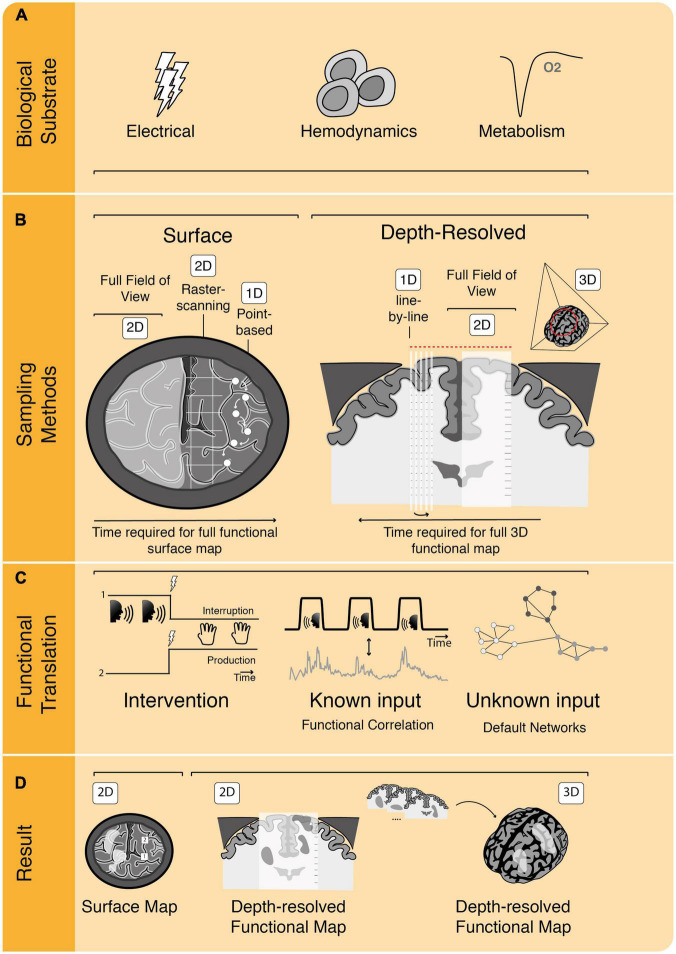
The process of functional image acquisition. **(A)** Functional imaging techniques rely on detection of one or more underlying biological substrates which serve as indicators of functional activity. **(B)** Roughly, techniques can be subdivided in surface and depth-resolved, penetrative techniques, which either build their full field of view at once (in 2D or 3D) or using subsamples, such as point-based, raster-based or line-by-line scanning. **(C)** To translate from acquired data to functional information, techniques rely on one or more of three methods. They can be intervention-based, probing the brain by interrupting or producing certain behavior. They can rely on known input patterns of pre-defined functional tasks to use for functional correlation or they can use functional connectivity analyses without known inputs (i.e., determining resting-state or default networks). **(D)** Once functional analyses have been performed, functional maps are displayed as, e.g., 2D surface maps or 2D/3D depth-resolved functional maps.

Visualization of these different biological substrates for neuronal activity can take different forms, including 2D-heat maps or full 3D-renderings of the brain and its activated areas. To go from detection of neuronal activity or its proxies to an actual image of functionality, different strategies are employed by different techniques, summarized in Figure 2.

First, all functional imaging techniques rely on sampling of one or more of the previously mentioned biological substrates which together drive an intricate cascade of electrical, metabolic and hemodynamic events (Iadecola, 2017; Figure 2A). The exact biological substrates underlying each of these techniques will be discussed extensively in the next section. Second, there are different methods to sample functionally informative signal in the brain, varying from point or raster measurements to sampling the full craniotomy at once, such as in optical techniques (Figure 2B). Other techniques allow for depth-resolved penetration of brain tissue, which can be achieved by assembling multiple lines to create a 2D-subplane, by sampling a full 2D-subplane at once, or even 3D-volumes at once. Depth penetration is a characteristic inherent to the technique’s contrast mechanism: optical techniques generally have low penetration due to high scattering and absorption by tissue, whereas ultrasonic scattering is orders of magnitudes weaker, allowing for deeper penetration (Yao et al., 2014). Often there is a trade-off between a technique’s spatial resolution and its penetration depth. The spatial resolution of a technique concerns the physical dimensions that the technique’s smallest unit of measure represents. In the case of raster scanning, for example, this would concern the dimensions of the smallest unit of measure (pixel) which can be sampled to build the full image. Some techniques such as Laser Doppler Flowmetry (LDF), are penetrative and able to sample volumes of the brain, without actual depth-resolution, meaning that they are not “depth-resolved” as signal cannot be spatially discerned along their depth-axis of sampling.

Additionally, there are roughly three different methods to translate these signals to functionally meaningful images: (1) by correlating the sampled signal to a pre-defined functional task pattern (presented to the patient), (2) by uncovering inherent patterns of default network connectivity within the brain signal *without* a known input (resting-state imaging), and (3) by electrically probing the brain to interrupt or produce functionality (Ritaccio et al., 2018; Figure 2C). In all three cases, the functional signal needs to be sampled over time, with the density of sampled data points within a certain amount of time defining the technique’s temporal resolution. How long a sample needs to be in order to be able to determine functionality (i.e., the acquisition time) is also technique-dependent, which together with the spatial sampling methods described above, can have a great impact on both data rates and total data size.

Finally, the results of the functional translation can be presented in different ways. A majority of these functional techniques work with functional maps superimposed over morphological images of tissue (Figure 2D). In some cases, this is restricted to the 2D brain surface only, while in other techniques volumes of the full brain are produced. The time it takes to acquire and visualize a single functional dataset determines whether the technique can be considered to be “real time,” or in other words, whether the technique is fast enough to use in the OR for surgical decision-making. Techniques which rely on 3D-sampling of functional brain volumes, will be inherently faster compared to subplane-based techniques. We will make these differences in visualization of functionality explicit for each technique throughout the manuscript and will come back to this topic in the discussion. Below, we will first categorize and describe the functional techniques based on their underlying biological substrate.

## 4. Electricity-based techniques

The electrical activity produced by neurons serves as a direct measure of neuronal activity (Barnett and Larkman, 2007), which can be sampled at different spatiotemporal scales, ranging from a single-unit or multiple thousands of neurons at once using microelectrode arrays (MEAs) (Hong and Lieber, 2019). Current technical efforts for electricity-based techniques seem to focus on further miniaturization of these electrodes and increase in electrode density. As an example, Neuralink recently introduced so-called micron-scale “threads,” with thousands of electrodes per array which can be inserted into the brain using a neurosurgical robot (Musk and Neuralink., 2019). Note that in this paper we will not include this and other techniques such as stereotactic EEG (sEEG) (Lachaux et al., 2003) which facilitate direct neuronal recording *only* through such invasive methods.

A non-invasive alternative which *does* allow for imaging is Electrocorticography (ECoG), where local field potentials (LFPs) are sampled using cortical grids placed over the exposed surface of the brain (Jeremy Hill et al., 2012). The size and distribution of the electrodes on the ECoG-grids determines the spatial resolution (subcentimeter), with penetration depths up to a centimeter. ECoG’s extra-operative counterparts are Magnetoencephalography (MEG) and Electroencephalography (EEG). MEG is a non-invasive technique which uses highly sensitive scalp-detectors to measure minuscule magnetic fields created by intra-cellular current flow of active neurons (Mäkelä et al., 2006; Stufflebeam, 2011). MEG’s spatial resolution is excellent (several millimeters), but it is not widely available given its high costs and low signal-to-noise ratio (Mäkelä et al., 2006; de Hoyos et al., 2018). EEG is dependent on the distribution of extracellular volume currents (Mäkelä et al., 2006; Stufflebeam, 2011). Its spatial resolution (centimeters) is much less than that of MEG, as electrical field potentials are more perturbed by the skull (Mäkelä et al., 2006; Stufflebeam, 2011).

Electrocortical stimulation mapping (ESM) [also known as Direct Electrical Stimulation (DES)] relies on *active* production (*positive responses*) or interruption (*negative responses*) of neuronal activity through interaction with the brain using bipolar stimulating forceps while the patient is awake and performing functional tasks (Borchers et al., 2011). In case of disruption of the patient’s performance during stimulation, that specific area of the cortex is marked with numbered cut-outs. The estimated spatial resolution of ESM is below one centimeter (Pouratian et al., 2004). The duration of the full mapping effort and can take multiple minutes (Ritaccio et al., 2018). ESM is thought to cover a volume of several millimeters of tissue without actual depth resolution, depending on stimulation amplitude (Pouratian et al., 2004). The underlying physiology of ESM remains poorly understood, and behavioral effects of different stimulation sites across and even in the same individual seem highly variable (Borchers et al., 2011). Literature therefore warns us to not be “*deluded by the obvious fascination of direct access to the human brain”* which ESM offers (Borchers et al., 2011). Nevertheless, ESM holds the potential to identify brain regions which might be *necessarily* involved in a particular functional task, rather than areas that are “only” correlated. To give an example: if during a language tasks a certain stimulation site leads to complete speech arrest of the patient, a surgeon will often spare that brain region during tumor removal as it seems highly involved in speech. This is fundamentally different from the other correlation-based imaging techniques discussed here, where the level of involvement or the necessity of a brain region for certain function behavior seems more elusive. ESM’s extra-operative counterpart is Transcranial Magnetic Stimulation (TMS), which relies on the principle of electromagnetic induction (Tarapore et al., 2016) to facilitate activation- or inhibition-based functional mapping. TMS is able to reach a spatial resolution of one centimeter (Sliwinska et al., 2014) with penetrations depths of centimeters (Deng et al., 2014).

The contact-free, optical techniques of Calcium-imaging (Ca-i) and Voltage imaging [V-i, including Voltage Sensitive Dye imaging (VSD-i) and Genetically Encodable Voltage Indicator imaging (GEVI-i)] exploit artificial dyes or indicators as measures of changes in membrane potential or the flux of calcium, respectively. The fluorescence microscope and camera dictate the spatial resolution (<50 μm), temporal resolution (msec), and field of view. Like most other optical imaging techniques, penetration depths are limited (<500 μm to max. millimeter-range) (Carlson and Coulter, 2008; Ferezou et al., 2009; Xu et al., 2017; Vogt, 2019). So far both techniques have only been described in animal experiments, limited by many challenges, not the least of which being the need for invasive injections or genetic modification to introduce dyes or indicators (Grienberger and Konnerth, 2012; Xu et al., 2017).

## 5. Hemodynamics-based techniques

In Laser Doppler Imaging (LDI) a brain region is illuminated by laser light, which is reflected back and picked up by a camera, making the technique contact-free (Raabe et al., 2009). Laser Doppler Flowmetry (LDF) is the one-dimensional equivalent of LDI, where laser light is delivered to the tissue using a multimode fiber and back-scatted photons are detected using another multimode fiber, both often integrated into a single fiber-optic probe (Frerichs and Feuerstein, 1990; Rajan et al., 2009). The light scattered back from RBCs will have undergone a Doppler-based frequency shift, which is proportional to the RBC velocity. As such, LDI measures CBF. LDI is known for its high temporal resolution (milliseconds), reasonable spatial resolution (micro- to millimeter), but limited penetration depth (millimeter) dependent on the diameter of the laser beam (Raabe et al., 2009; Rajan et al., 2009). As demonstrated by Raabe et al. (2009), LDI would be particularly suitable to integrate into conventional surgical microscopes using superimposed functional maps, which could “*considerably shorten the time required for mapping the exposed brain*.”

Laser speckle contrast imaging (LSCI) emits light on the tissue to create a random interference pattern known as speckle (Briers et al., 2013; Heeman et al., 2019), which can be detected by a camera (Boas and Dunn, 2010). When the light scatters of moving particles such as RBCs, this will cause temporal fluctuations in the interference, and therefore lead to intensity or contrast variations in the speckle pattern, from which we can infer CBF (Boas and Dunn, 2010). Similar to LDI, LSCI is limited to penetration depths of millimeters (Senarathna et al., 2013). The spatial resolution can go down to <50 μm (Senarathna et al., 2013), depending on camera resolution and the minimum speckle size, which is directly proportional to the laser wavelength (Boas and Dunn, 2010). Both LDI and LSCI cover the full superficial cortical surface revealed by the craniotomy, while remaining contact-free. LSCI has been tested in an experimental intra-operative setting for neurovascular as well as neuro-oncological procedures (Hecht et al., 2009; Klijn et al., 2013).

Optical Coherence Tomography (OCT) is the optical, contact-free analog of ultrasound imaging and uses low-coherence, broad bandwidth light: a light beam from a source is split in two, where one beam is considered the “reference” beam and the other the “sample” beam, sent out in the tissue of interest (Huang et al., 1991). Dependent on the refractive index of the tissue and size of scattering particles, a fraction of the light is back scattered. The reference beam, recombined with the backscattered “sample” beam, produces an interference pattern which provides the information needed to resolve structures at every depth along the path of the beam (Huang et al., 1991; Dayani et al., 2009; Baran and Wang, 2016; Wachulak et al., 2018; Almasian et al., 2019; Ughi et al., 2020). When combining this structural information with information on blood flow and volume gained from the above-described Doppler signal, the functional alternative dOCT is derived. OCT can reach micrometer precision (Wachulak et al., 2018), with a penetrative depth of millimeters. Its temporal resolution depends on the scanning volume, but can be as low as several milliseconds (Gabriele et al., 2010; Yuan et al., 2017; Wachulak et al., 2018). No report of intra-operative neurosurgical application of dOCT has been published.

Functional Ultrasound (fUS) is a relatively new technique which exploits a high-frame-rate (HFR) acquisition scheme to boost the sensitivity of Doppler ultrasound (Macé et al., 2011). The gained sensitivity allows for detection of subtle changes in motion caused by moving RBCs, facilitating Power Doppler visualization of hemodynamics in the brain’s microvasculature (Macé et al., 2011), with spatial resolutions in the submillimeter-range. Depending on the ultrasound frequency used, penetration depths of multiple centimeters are conventional (Imbault et al., 2017; Soloukey et al., 2020; Baranger et al., 2021). Several approaches to performing fUS in 3D or even 4D have also been reported in literature (Rau et al., 2018; Rabut et al., 2019; Brunner et al., 2020). Most approaches include volumetric fUS by acquiring and combining multiple scans using 2D-transducer arrays (Brunner et al., 2020). In all cases, fUS requires that acoustic contact is maintained with the brain tissue using ultrasound gel or saline solution. Literature reports two successful intra-operative applications of fUS during awake craniotomy functional mapping (Imbault et al., 2017; Soloukey et al., 2020).

## 6. Metabolism-based techniques

As the energetic demands of neuronal activity are met by the production of ATP, metabolites are consumed and produced, acting as indicators of neuronal activity (Viswanathan and Freeman, 2007). Intrinsic Functional Fluorescence imaging (IFF-i) exploits the fact that specific metabolic molecules such as oxygenated mitochondrial Flavoprotein present with endogenous fluorescence, which can be detected by a CCD-camera. Like most optical techniques, IFF-i is considered to have good spatial resolution (<500 μm), but with limited penetrative depth (millimeter) (Husson and Issa, 2009). Literature reports no intra-operative applications of IFF-i.

Similarly, Optical Intrinsic Signal Imaging (OISI) (Haglund et al., 1992) relies on the contact-free detection of small, intrinsic signal, which is thought to consist of (1) changes in local CBV, (2) changes in oxygen saturation level of hemoglobin and, (3) changes in light-scattering due to cortical activation (Sato et al., 2017). The first element could justify categorizing OISI not only as a metabolism-based but also a hemodynamics-based technique. The initial regional dip in oxygenated hemoglobin forms a major component of the OISI signal, the temporal resolution of which is mostly dictated by the CCD-camera (milliseconds). OISI performs relatively well in spatial resolution (submillimeter) but has limited penetrative depths. Literature reports a number of in-human, intra-operative applications of OISI during oncological as well as epilepsy-related neurosurgical procedures (Haglund et al., 1992; Cannestra et al., 1996, 2000; Schwartz et al., 2004; Nariai et al., 2005; Prakash et al., 2009; Meyer et al., 2013; Sobottka et al., 2013; Sato et al., 2017; Oelschlägel et al., 2020).

Infrared Thermography (IT) or thermo encephalography detects infrared radiation as generated during local energy production by using a contact-free thermoviser camera (Shevelev et al., 1993; Gorbach et al., 2003). Although CBF-changes have a large component in local tissue temperature increase, the technique’s actual sensitivity for functional signal remains controversial (Hoffmann et al., 2018). A single 2D-image can be acquired within several milliseconds and is only a superficial snapshot of the brain cortex, as IT has a depth penetration of millimeters. The spatial resolution of IT is reasonable (submillimeter). IT has been applied in an intra-operative setting on several occasions by integrating the imaging technique into conventional surgical microscopes (Shevelev et al., 1993; Gorbach et al., 2003; Hoffmann et al., 2018).

Photo-Acoustic Imaging (PAI) and Functional near-infrared spectroscopy (fNIRS) are optical techniques which rely on the different absorption spectra of (de-)oxygenated and total hemoglobin (HbO, HbR, and HbT). In fNIRS, a detector is placed several centimeters away from a light source, receiving diffused light which contains information on cerebral hemoglobin concentration of the tissue within the light path (Boas et al., 2014; Chiarelli et al., 2017). fNIRS has a centimeter-millisecond spatiotemporal resolution, with a depth penetration of several centimeters, without actually being depth-resolved. Because the NIR-spectrum light attenuation in the skull is relatively low, fNIRS could even facilitate transcranial imaging (Jobsis, 1977; Ferrari and Quaresima, 2012; Chiarelli et al., 2017; Yücel et al., 2017). fNIRS has been used both in an intra-operative (Fukuda et al., 2015; Sato et al., 2016; Qiu et al., 2019) as well as extra-operative context using a wearable device (Pinti et al., 2015).

In PAI, optical excitation is combined with acoustic detection (Wang and Gao, 2014; Cao et al., 2017; Ovsepian et al., 2017; Ugalde, 2018). After illuminating the tissue with a laser pulse, chromophores such as RBCs absorb the photonic energy depending on the oxygenation-status of their hemoglobin which leads to a transient, localized rise in temperature (Wang and Hu, 2012; Cao et al., 2017). Due to thermoelastic expansion, the absorbing RBCs generate an acoustic wave picked up by an ultrasound transducer. Because PAI does not rely on the light to return to a detector it can reach significant penetrative depths (centimeters). The technique’s spatial resolutions ranges from <10 μm with low penetration depths (1 mm) functional Photoacoustic Microscopy (fPAM) (Wang and Gao, 2014), to penetration depths of several centimeters [functional Photoacoustic (Computed) Tomography (fPA(C)T)] to allow for deep, volumetric imaging (Gottschalk et al., 2015; Tang et al., 2016). Similar to fUS, PAI requires continuous acoustic contact. Recently, the first successful in-human application was described in literature using a new 3D fPACT system, validated to fMRI (Na et al., 2022).

In the extra-operative realm, transcranial, metabolic techniques such as Single-Photon or functional Emission Computed Tomography (SPECT/fPET) and fMRI are used. In fMRI, we can discriminate between blood signal depending on its degree of oxygenation (BOLD), which serves as an indirect measure of the level of neuronal activity. The BOLD-signal has a multiple second latency as compared to the original oxygen dip detected by OISI and involves the previously mentioned process of functional hyperemia (Logothetis et al., 2001). fMRI in its clinical form has a spatial resolution in the millimeter range, a temporal resolution of 1–2 s, and is often task-based (tb-fMRI) (Logothetis, 2008). In terms of pre-surgical planning, tb-fMRI has an established role in determining, e.g., language or sensorimotor lateralization (Agarwal et al., 2019). Acquisition time for a single functional task usually consists of several minutes, with total time spent in the scanner being close to an hour in our clinical institution. In experimental settings, resting-state fMRI (rs-fMRI) has been used to identify mostly language and motor-related brain areas (Hacker et al., 2019). A limited number of (awake) intraoperative fMRI [(a)ifMRI] cases have been reported (Lu et al., 2013), where susceptibility artifacts and disruption of surgical workflow make the technique unfavorable.

Single-photon emission computed tomography and fPET are based on the detection of radioactivity which is emitted by radioactive tracers such as H_2_^15^O to examine CBF (Berger, 2003; Li et al., 2020). During their metabolism, these radioactive tracers ultimately emit gamma rays which are subjected to “coincidence detection,” simultaneous detection by two opposing detectors placed in a ring surrounding the patient. The spatial resolution (millimeter) is determined partially by these two detectors, which define the line of response along which the original emission took place (Berger, 2003; Li et al., 2020). In terms of temporal resolution, PET has a poor performance, in the minute-range, limited both by the technique itself as well as the required time needed for the metabolism of the radioactive molecule (Berger, 2003; Li et al., 2020). A full acquisition can take up to an hour. SPECT differs from PET only in that it uses gamma (single photon) emitters. Therefore, there is no information about the direction of the incoming photon and the spatial resolution (>1 cm) is lower than that of PET (Rahmim and Zaidi, 2008).

**FIGURE 3:**
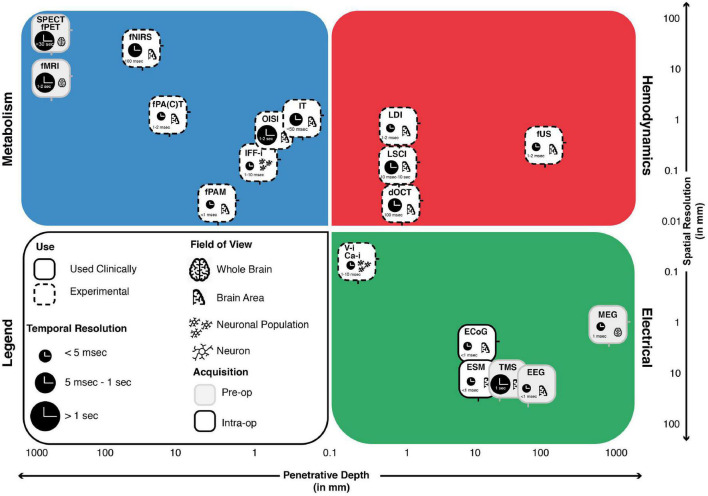
The landscape of intra-operative functional imaging techniques and their respective technical characteristics. Metabolism-, Electrical- and Hemodynamics-based techniques are placed within a grid spanning spatial resolution (*y*-axis) and penetrative depth (*x*-axis). The techniques field of view, current status of use, intra-operative applicability and temporal resolution are also taken into considerations. Many of the techniques illustrate a trade-off between penetrative depth and spatial resolution, with high penetration, lower resolution techniques such as SPECT and fPET on the one end and low penetration, high resolution techniques such as V-i and Ca-i on the other end of the spectrum. Unique positions within the domain are taken up by techniques such as MEG, which is able to combine full brain penetration with good spatiotemporal resolutions. A similar position is taken up by fUS, which combines sufficient penetration depths with exceptional spatiotemporal resolution, while showing actual intra-operative applicability. ECoG, electrocorticography; EEG, electroencephalography; MEG, magnetoencephalography; ESM, electrocortical stimulation; Ca-i, calcium-imaging; V-i, voltage-imaging; TMS, transcranial magnetic stimulation; NVC, neurovascular coupling; PAI, photo-acoustic imaging; fNIRS, functional near-infrared spectroscopy; dOCT, Doppler optical coherence tomography; fUS, functional ultrasound; LDI, laser Doppler imaging; LSCI, laser speckle contrast imaging; RBC, red blood cells; NMC, neurometabolic coupling; IFF-I, intrinsic functional fluorescence-imaging; IT, infrared thermography; OISI, optical intrinsic signal imaging; fMRI, functional magnetic resonance imaging; fPET, functional position emission tomography; SPECT, single-photon emission computed tomography; Pre-op, pre-operatively available only; Intra-op, intra-operatively available.

All technical characteristics of above-mentioned techniques are summarized in the schematic overview in Figure 3. More information on these techniques can also be found in the (Appendix B) or on www.openneurosurgery.com.

## 7. Discussion

Starting from the premise that real-time functional brain imaging has the potential to improve a neurosurgeon’s intra-operative decision-making, we compared and contrasted the biological and technical characteristics of all clinical and experimental techniques which show potential for real-time functional imaging of the exposed brain. This paper is the first to focus specifically on the exposed brain, with the ultimate aim of providing a paradigm from which to understand or categorize these techniques, in contrast to other reviews on functional techniques available in literature (De Witte et al., 2015; Ottenhausen et al., 2015; Hu et al., 2018; Sagar et al., 2019; Azad and Duffau, 2020). We regard the exposed brain not to be just an *opportunity* for such functional imaging applications, but also a *necessary* condition for the majority of these techniques, which are otherwise blocked or limited by the presence of the skull.

The actual applicability of these techniques on “an exposed brain,” however, is highly context dependent. Functional interrogation of the brain involves time and computational power, both limited in the surgical context. An fPAM-based functional map might be detailed but requires time-consuming raster-scanning to cover the full craniotomy. Camera-based techniques like OISI will map the full craniotomy during one functional task, leaving time to perform more tasks. Similarly, volumetric functional maps can be insightful, but data rates that come with depth-resolved, high-resolution images will prove challenging. If the 3D-volume is not instantaneous but consists of multiple stacked 2D-acquisitions, actual intra-operative application is even less feasible as each 2D-repetition will take a full task duration. A technique’s sampling scheme as summarized in Figure 2 dictates the time to acquire and update a functional map and as such, whether the technique is “real-time,” meaning fast enough to use for surgical decision-making. It therefore makes sense that ESM with its limited technical characteristics, remains widely used given its ease of use and instant functional feedback.

**FIGURE 4:**
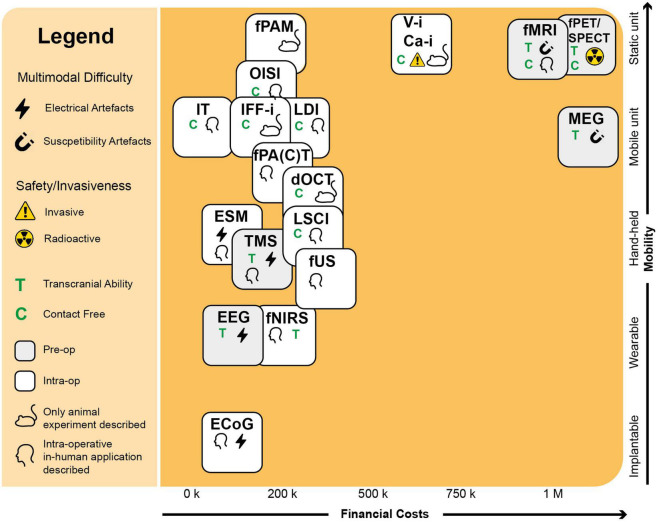
Requirements for the intra-operative context. Here we see an overview of the functional neuro-imaging landscape, drawn up using parameters with a clinical and intra-operative relevance. In the upper right corner, we see a group of three pre-operative techniques (MEG, fMRI, fPET/SPECT) with high costs and limited mobility, partially explaining their limited intra-operative applicability. Also the contact-level needed for the technique to function can dictate the technique’s intra-operative applicability. Optical techniques such as OISI and IT, make use of stand-alone cameras or integrated versions into conventional surgical microscopes, which does not require any substantial interruption of the surgical flow. Techniques such as fUS or PAI come in mobile, hand-held acquisition units, which do not take up much space in the operating room. A technique’s multimodal potential is also important and can be complicated by electrical or susceptibility artifacts. Finally, the level of invasiveness can severely determine its clinical success. As Ca-i and V-i require introduction of alien fluorescent markers, their clinical applications is more complicated than techniques such as OISI and IFF-i which rely on intrinsic fluorescence. ECoG, electrocorticography; EEG, electroencephalography; MEG, magnetoencephalography; ESM, electrocortical stimulation; Ca-i, calcium-imaging; V-i, voltage-imaging; TMS, transcranial magnetic stimulation; NVC, neurovascular coupling; PAI, photo-acoustic imaging; fNIRS, functional near-infrared spectroscopy; dOCT, Doppler optical coherence tomography; fUS, functional ultrasound; LDI, laser Doppler imaging; LSCI, laser speckle contrast imaging; RBC, red blood cells; NMC, neurometabolic coupling; IFF-I, intrinsic functional fluorescence-imaging; IT, infrared thermography; OISI, optical intrinsic signal imaging; fMRI, functional magnetic resonance imaging; fPET, functional position emission tomography; SPECT, single-photon emission computed tomography; Pre-op, pre-operatively available only; Intra-op, intra-operatively available.

The ideal surgical technique would be mobile, low-cost, non-invasive with high depth-penetration and spatiotemporal-resolution, whole-brain transcranial imaging opportunity and fast acquisition times. Techniques such as fUS (Soloukey et al., 2020) and functional Photoacoustic Computed Tomography (fPACT) (Na et al., 2022) come close, as they combine mobile units with unconventionally high spatiotemporal resolutions and penetrative depth (Figure 4). However, these techniques are not transcranially compatible as of yet. If we could use these techniques both intra- and extra-operatively, time for surgical planning could be significantly extended, as well as the opportunity for post-operative monitoring once skulls covers the brain again. Additionally, if techniques like fUS (Soloukey et al., 2020) and fPACT (Na et al., 2022) can be translated to the transcranial setting, we will have a new arsenal of highly suitable techniques to apply in Brain Computer Interfaces (BCI) (Smalley, 2019). The first successful examples of fUS-based BCIs in non-human primates have been reported recently (Norman et al., 2021; Griggs et al., 2022). However, the challenge to move to a transcranial modality is harder for some techniques than others. While fNIRS is known in transcranial form (Curtin et al., 2019), for optical techniques such as dOCT with limited penetrative depth, it is hard to imagine the same. For fPACT and fUS, however, transcranial might be a possible future frontier to cross if we can find ways to correct for the skull’s attenuation.

It is important to realize that in the surgical context functional imaging often equals functional *delineation* of, e.g., a tumor, epileptogenic zone or deep brain nucleus. The surgical goal of *delineating* a tumor, in practice means maximum removal of local tissue which is “safe,” not directly *eloquent* or *necessarily* involved in functional behavior. This has important consequences for how we think about functional imaging techniques and what they should achieve in the surgical setting. Techniques such as ESM, although spatially crude, could still help identify *necessary* brain regions based on direct interruption of behavior during functional tasks. However, we must realize that we cannot interrogate or interrupt all functionalities in an intra-operative setting, meaning that these techniques often only take into account very crude and simplified functional tasks such as finger tapping or word repetition. That is assuming the optimal circumstances of an awake surgery where a patient *does* actively and correctly perform tasks, in contrast to anesthetized patients who can only be subjected to limited passive tasks (see Appendix C for examples of functional tasks in awake vs. anesthetized context). Awake craniotomy requires a team of highly trained individuals, including a neuropsychologist or clinical linguist, who is familiar with the patient’s baseline functional performance, in order to correctly interpret intra-operative changes. In reality, the awake setting is often far from optimal, given limited time for mapping, as well as limited ability for the head-fixated patient to physically perform tasks. Given the emotional context of the surgical procedure, as well as the per-operative anesthesia and analgesics that are administered, the intra-operative cognitive capacity of the awake patient is also limited, clouding behavioral interpretation as surgery progresses. If we were to use real-time, depth-resolved techniques such as fUS, we could move from task-based interruption to continuous monitoring during, e.g., natural speech as the surgery progresses. However, here too much of the tissue might be deemed “functional,” even parts of the brain which might not be *necessary* for certain functional behaviors, leading to *too* conservative resection margins. What further complicates this trade-off is the fact that both the mechanisms underlying ESM (Borchers et al., 2011), as well as the definition of what would count as a *necessary* brain region, are up for debate. To truly solve this dilemma, we need to expand our neuroscientific understanding of brain functionality in general, which something we can do in the intra-operative context as well, as will be explained below. If our surgical goal would be to remove *just* tumor tissue, we would like to define its border as accurately as possible, where techniques such as Raman-spectroscopy would be suitable (Hollon et al., 2016). Finding the border of a tumor, avoiding eloquent areas or delineating functional areas, are three different goals that require different techniques with different surgical outcomes.

Finally, we want to emphasize how functional imaging in the surgical context is valuable for more than just surgical outcomes. As described by the Nature News Feature *Neuroscience: Opening Up Brain Surgery*, “*Neurosurgery can contribute to neuroscience by giving a glimpse into the mind, a rare window into the brain”* (Abbott, 2009). Similarly, *Science* recently featured a piece titled *Window of Opportunity*, where the authors argue that through conventional neurosurgical practice “*we can essentially gain access to the very basic neural mechanism of the human condition”* (Servick, 2022). Neurolinguistics is a great example of a field mostly dependent on limited techniques such as EEG and fMRI. fUS has demonstrated to be a useful tool to intra-operatively capture–with unprecedented detail—language-related activation in the brain of an awake patient (Soloukey et al., 2020). The future holds more: we can actively interact with the exposed brain by probing or temporarily interrupting language using ESM, ultimately translating individual data-points to group-level conclusions about brain functionality. We can study the effect of tumor invasion on language production and in real time image return of linguistic skill as tumor load is reduced intra-operatively. The possibilities to study cognitive phenomena in the OR seem endless, especially using techniques still limited in transcranial applicability, but with high imaging potential. What is highly encouraging in this effort: literature reports a clear willingness of patients and their families faced with for example Traumatic Brain Injury (TBI) to participate in and support scientific research (Hasan et al., 2019).

The human brain is exposed for a limited amount of time in a neurosurgical procedure, during which imaging data can provide functional maps to navigate the brain. For lower-risk tumor resections, an initial superficial map to determine point of entry might be enough to ensure surgical safety. The remainder of the procedure could be navigated using anatomy, tissue morphology and frankly, experience. Here, camera-based techniques such as OISI or LSCI are fast and accurate alternatives to conventional point-by-point ESM. For procedures in highly eloquent areas, we need continuously updated, depth-resolved functional maps as we navigate deeper in the brain. Here, techniques such as fUS and fPACT have potential, provided that we can handle their high data rates in the OR. While different neurosurgical procedures ask for different functional maps to navigate surgical territories, neuroscience will benefit from all these maps. In the surgical context we can combine healthy volunteer studies, lesion studies and even reversible lesion studies in in the same individual. Ultimately, individual cases will build a greater understanding of human brain function, which in turn will improve neurosurgeons’ future navigational efforts.

## Author contributions

SS and PK developed the concept, designed the manuscript, and wrote the first draft. All authors were involved in critically revising the first draft and approved the final version of the manuscript.
