# Supplementary material for: Functional imaging of the exposed brain
Source: Front Neurosci. 2023 Feb 9;17:1087912. doi: 10.3389/fnins.2023.1087912 (PMC9947297; doi:10.3389/fnins.2023.1087912)
Supplement: Supplementary file 1 [file Data_Sheet_1.docx]

**Appendix A – Glossary**

| 4D-data | Continuously updated 3D-data, allowing for e.g. detection of movement in 3D-images |
| --- | --- |
| Acoustic contact/coupling | The contact between ultrasound transducer and brain tissue which is necessary to facilitate the transmission and receival of acoustic signal. This can be achieved through e.g. ultrasound gel or water |
| Awake Craniotomy | Procedure where a patient is operated on the brain while being awake, facilitating communication, the performance of functional task etc. |
| Biological Substrate | The biological substrate deals with the underlying physiological process which is used as a measure of functionality by each of the techniques. |
| Blood dynamics | Changes in CBV (the volume of blood in a volume of brain) and changes in CBF (the speed with which a volume of blood perfuses a volume of brain) |
| Blood dynamics-based Functional Imaging Techniques | Imaging techniques which are based on sampling of one or more of the blood-dynamics related biological substrates involved in the process of neurovascular coupling |
| Brain-Computer Interface | A computer-based system that interfaces with the central nervous system to acquire signals, analyzes them, and translates them into commands that are relayed to an output device to perform a desired action. |
| Brain Functionality | The brain tissue’s ability to produce specific functional tasks or states, such as moving the hand or repeating a word. |
| Chromophore | Molecules in the tissue that absorb a particular wavelength of light |
| Coincidence Detection | Simultaneous detection by two opposing detectors |
| Depth of penetration | The depth of the brain tissue which can be reached by a penetrative technique. |
| Depth-resolved/Depth-resolution | The possibility to spatially discern the signal along the depth-axis |
| Doppler-effect | The change in frequency of a wave caused by motion, e.g. movement of a red blood cell |
| Ease of use | The extent to which a technique can be applied without previous training or expertise |
| Electrical after-discharges | Electrical stimulation (ESM)-induced spread of current which may lead to 1) activation of adjacent functional areas, represented by clinical manifestations not representative of the stimulus site or 2) epileptic seizure elicitation |
| Electrical Artifacts | Artifacts caused due to external injection of electrical current into the tissue |
| Electrical Functional Imaging Techniques | Imaging techniques which are based on (direct) electrical sampling |
| Eloquent Brain Area | Area of the brain that speaks to readily identifiable neurological function and, if injured, result in a disabling neurological deficit |
| Exposed Brain | (Part of) the brain with skull and dura removed |
| Field of View | The field of view refers to the extent of the desired tissue coverage which can be achieved by the imaging technique |
| Functional Delineation | The process of finding and visualizing the borders of a particular functional brain area in the brain |
| Functional Hyperemia | A regional increase of blood flow in response to local neural activation |
| Functional Imaging Technique | Any technique which has the ability to *visualize* functionality of brain tissue in a non-invasive manner |
| Functional Map | Visual representation of functional areas, often in the form of a color-coded map representing parameters such as coefficient of correlations, superimposed over a morphological or structural image |
| Functional Technique | Any technique which has the ability to detect functionality of brain tissue |
| Hemodynamics | The interplay of changes in blood dynamics (Cerebral Blood Volume (CBV), Cerebral Blood Flow (CBF)) and vascular dynamics (vessel diameter (vasoconstriction/vasodilatation)) |
| Imaging Duration | The amount of time necessary to generate the full functional volume of interest. This should be considered as the approximate time needed in the OR to acquire a functional map which can be used for surgical decision-making. |
| Interference | Interference is a phenomenon in which two waves superpose to form a resultant wave of greater, lower, or the same amplitude. |
| Invasiveness | The extent to which a technique 1) needs to penetrate the brain tissue to sample brain functionality to and/or 2) requires invasive practices for its functionality (e.g. administering a radioactive tracer for fPET). |
| Maximum Safe Tumor Resection | Maximal survival benefit by removal of tumor bulk with minimal risk of post-operative neurological deficit |
| Metabolic Functional Imaging Techniques | Imaging techniques which are based on sampling of one or more of the biological substrates involved in the process of neurometabolic coupling |
| Mobility | The extent to which the imaging technique itself or the technique’s acquisition unit can be moved. |
| Multimodal potential | The extent to which a technique can be used in concomitance with other techniques. Some techniques are inherently more difficult to combine with others, due to for example electrical or susceptibility artefacts. |
| Negative response (ESM) | Interruption of a functional task being performed during electrocortical stimulation mapping (ESM) |
| Neurometabolic Coupling | The mechanisms responsible for linking (electrical) neuronal activity to corresponding changes in consumption of metabolites such as oxygen and glucose |
| Neurovascular Coupling | The mechanisms responsible for linking (electrical) neuronal activity to corresponding changes in blood dynamics |
| Neurovascular Uncoupling | (Pathological) mechanisms due to which (electrical) neuronal activity is no longer coupled to corresponding changes in blood dynamics |
| Non-invasive technique | Technique which does not require physical penetration of brain tissue |
| Penetrative Depth | The depth to which an imaging technique can resolve functional acitivity in brain tissue |
| Photo-acoustic Imaging | Imaging modality which makes use of the photo-acoustic effect: laser pulses delivered to tissue cause transient thermoelastic expansion, and as such, ultrasonic emission which is detected by ultrasound transducers |
| Positive response (ESM) | Production of a response (i.e. language/motor movement) during electrocortical stimulation mapping (ESM) |
| Real-time functional imaging technique | An imaging technique which has close to no delays (instantaneous) when acquiring and producing functional images |
| Resting-State Imaging | Imaging based on uncovering inherent patterns of network connectivity within the brain signal *without* a known input |
| Sampling Method | The method used by a technique to sample the biological substrate of interest. This can be superficial sampling, or penetrative sampling, either with or without depth resolution. Sampling can be achieved of the full field of view at once, or by combining multiple subsamples. |
| Spatial Resolution | The spatial resolution of a technique concerns the physical dimensions that the technique’s smallest unit of measure represents. In case of images, this would concern the physical dimensions represented by a single pixel within the image. |
| Speckle | Speckle is the results of the interference of many waves of the same frequency which added together result in a wave with a randomly varying intensity |
| Structural Technique | Any technique which has the ability to visualize structure and/or morphology of brain tissue |
| Surgical Workflow | The course of the surgical intervention and the conventional steps in the surgical procedure |
| Susceptibility Artifacts | Artifacts caused due to differences in magnetic susceptibilities of tissues or materials. In MRI, this is characterized by geometric distortion, including brighter or darker areas. |
| Task-based Imaging | Imaging based on providing the brain with a known input (a functional task pattern) to correlate the sampled signal to |
| Temporal Resolution | The temporal resolution tells us with which frequency a technique is able to sample the biological substrate which underlies a technique within a unit of time. |
| Tomography | Imaging through section of tissue using any kind of penetrating wave |
| Transcranial Functional Imaging Technique | A technique which has the ability to visualize brain functionality through the skull |
| Vascular Dynamics | Vasoconstriction and vasodilatation, mediated by smooth muscle cells (SMCs) surrounding larger veins, arteries and smaller arterioles |
| Visual Presentation | The way in which the sampled information on brain functionality is presented. Depending on the technique, this can e.g. include 2D color maps or volumetric reconstructions of functional maps. |

**Appendix B – Supplementary Table 1. Table of Characteristics per Imaging Technique**

| **Technique** | **Biological Substrate** | **Sampling Method** | **Field of View** | **Functional Translation – *Intervention, Functional Task, Default Network***** | **(Potential) Applicability** | **Previous intra-operative in-human application?** | **Spatial Resolution** | **Depth of Penetration/ Depth resolved?** | **Temporal Resolution** | **Contrast Mechanism** | **Mobility** | **Transcranial Ability** | **Contact-free?** | **Multimodal Difficulty** | **Imaging duration** | **Costs** |
| --- | --- | --- | --- | --- | --- | --- | --- | --- | --- | --- | --- | --- | --- | --- | --- | --- |
| **Ca-i** | Electrical | Superficial | Neuronal population | All | Intra-op | No | <50 µm | <1 mm/No | msec | Dyes  *- Fluorescence* | Static Unit | No *(Yes in animals)* | Yes *Requires calcium-indicator* | N/A | Minutes | >500k-1M |
| **V-i** | Electrical | Superficial | Neuronal population | All | Intra-op | No | <50 µm | <1 mm/No | msec | Dyes  *- Fluorescence* | Static Unit | No *(Yes in animals)* | Yes *Requires voltage-sensitive dye* | N/A | Minutes | >500k-1M |
| **ECoG** | Electrical | Superficial | Cortex | All | Intra-op | Yes | <1 cm | 1 cm/No | msec | N/A | Implantable | No | No | Electrical artefacts | Minutes | <200k |
| **ESM** | Electrical | Superficial (Point-based) | Cortex | Intervention | Intra-op | Yes | <1 cm | 1 cm/No | < 1 sec | N/A | Hand-held | No | No | Electrical artefacts | Minutes | <200k |
| **TMS** | Electrical | Penetrative | Cortex | Intervention | Extra-op | No | <1 cm | 2-3 cm/ Poorly | < 1 sec | N/A | Hand-held | Yes | N/A | Electrical artefacts | Minutes | <200k |
| **EEG** | Electrical | Penetrative | Whole Brain | Functional Task/Default Network | Extra-op | No | 1-2 cm | 5-10 cm/Poorly | msec | N/A | Wearable | Yes | N/A | N/A | Minutes | <200k |
| **MEG** | Electrical | Penetrative | Whole Brain | Functional Task/Default Network | Extra-op | No | 1-2 mm | 1 m/Yes | msec | N/A | Static Unit *(Mobile units/ Wearable under development)* | Yes | N/A | Susceptibility artefacts | Minutes | > 1M |
| **Technique** | **Substrate** | **Sampling Method** | **Field of View** | **Functional Translation – *Intervention, Functional Task, Default Network***** | **(Potential) Applicability** | **Previous Intra-operative in-human application?** | **Spatial Resolution** | **Depth of Penetration/ Depth resolved?** | **Temporal Resolution** | **Contrast Mechanism** | **Mobility** | **Transcranial Ability** | **Contact-free?** | **Multimodal Difficulty** | **Imaging duration** | **Costs** |
| **fUS** | Blood | Penetrative | Lobe | All | Intra-op | Yes | <500 µm | 5-10 cm/Yes | msec | Doppler | Hand-held/ Mobile Unit *(Wearable in animals)* | No | No | N/A | Minutes | >200k-500k |
| **LDI** | Blood | Superficial | Cortex | All | Intra-op | Yes | 1 mm | 1-5 mm/No (although path length-resolved LDF has been described) | msec | Doppler (scattering) | Mobile unit (LDF also available in hand-held form) | No | Yes | N/A | Minutes | >200k-500k |
| **LSCI** | Blood | Superficial | Cortex | All | Intra-op | Yes | <50 µm | 1-2 mm/No | msec – sec | Doppler (scattering) | Hand-held | No | Yes | N/A | Minutes | >200k-500k |
| **dOCT** | Blood | Superficial | Cortex | All | Intra-op | No | <50 µm | 1-2 mm/Yes | msec | Doppler (scattering) | Hand-held | No | Yes | N/A | Minutes | .>200k-500k |
| **Technique** | **Substrate** | **Sampling Method** | **Field of View** | **Functional Translation – *Intervention, Functional Task, Default Network***** | **(Potential) Applicability** | **Previous Intra-operative in-human application?** | **Spatial Resolution** | **Depth of Penetration/ Depth resolved?** | **Temporal Resolution** | **Contrast Mechanism** | **Mobility** | **Transcranial Ability** | **Contact-free?** | **Multimodal Difficulty** | **Imaging duration** | **Costs** |
| **fPET** | Metabolic | Penetrative | Whole Brain | Functional Task/Default Network | Extra-op | No | 5-10 mm | 1 m/Yes | sec-min | Radiotracer | Static Unit | Yes | N/A *Radioactive tracer* | N/A | Minutes-hour | > 1M |
| **SPECT** | Metabolic | Penetrative | Whole Brain | Functional Task/Default Network | Extra-op | No | 1-2 cm | 1 m/Yes | sec-min | Radiotracer | Static Unit | Yes | N/A *Radioactive tracer* | N/A | Minutes-hour | > 1M |
| **fMRI** | Metabolic (can also detect blood flow) | Penetrative | Whole Brain | Functional Task/Default Network | Extra-op *(except for i-fMRI)* | Yes | 1-5 mm | 1 m/Yes | sec | BOLD | Static Unit | Yes | N/A | Susceptibility artefacts | Minutes-hour | > 1M |
| **fNIRS** | Metabolic (can also detect blood flow) | Penetrative | Cortex | All | Intra-op | Yes | 1-2 cm | 2-3 cm/No | msec | Absorption | Wearable | Yes | No | N/A | Minutes | <200k |
| **fPA(C)T** | Metabolic (can also detect blood flow) | Penetrative | Cortex | All | Intra-op | No | <1mm | 1-3 cm/Yes | Msec-sec | Absorption | Mobile Unit *(Wearable in animals)* | No | No | N/A | Minutes | >200k-500k |
| **fPAM** | Blood | Superficial | Cortex | All | Intra-op | No | <50 µm | 2-3 mm/Yes | msec | Absorption | Static Unit | No | No | N/A | Minutes | >200k-500k |
| **IFF-I** | Metabolic | Superficial | Neuronal population | All | Intra-op | No | <500 µm | 1-2 mm/No | msec | Absorbance | Static Unit | No | Yes | N/A | Minutes | <200k |
| **OISI** | Metabolic | -Superficial -Full field of view at once | Cortex | All | Intra-op | Yes | <1 mm | <1 mm/No | msec | scattering | Mobile unit | No | Yes | N/A | Minutes | <200k |
| **IT** | Metabolic | Superficial | Cortex | All | Intra-op | Yes | <1 mm | <1 mm/No | msec | Radiation | Mobile unit | No *(Yes in animals)* | Yes | N/A | Minutes | <200k |

**Intra-op = Intra-operative, Pre-op = Pre-operative, Ca-i = Calcium-imaging, ECoG = Electrocorticography, ESM = Electrocortical Stimulation Mapping, EEG= Electroencephalography, MEG = Magnetoencephalography, TMS = Transcranial Magnetic Stimulation, V-i = Voltage-imaging, fNIRS = functional Near Infrared Spectroscopy, fUS = functional Ultrasound, LDI = Laser Doppler Imaging, LDF = Laser Doppler Flowmetry, LSCI = Laser Speckle Contrast Imaging, dOCT = Doppler Optical Coherence Tomography, PAI = Photo-acoustic Imaging, fPAM = functional Photo-acoustic Microscopy, fPA(C)T = functional Photo-acoustic (Computed) tomography, fMRI = functional Magnetic Resonance Imaging, (i)-fMRI = intraoperative functional Magnetic Resonance Imaging, fPET = functional Positron Emission Tomography, IFF-i = Intrinsic Functional Fluorescence Imaging, IT = Infrared Thermography, OISI = Optical Intrinsic Signal Imaging, SPECT = Single-photon Emission Computed Tomography, N/A = non-applicable.
** Refers to the terminology used in Figure 2 of the main manuscript. References to literature discussing above-mentioned techniques can be found in the main manuscript.*

**Appendix C – Supplementary Table 2. Overview of intra-operative functional ESM-tasks during awake vs anesthetized craniotomy for tumor removal**

| **Awake** | **Task (examples)** | **Eloquent Area(s)** | **Possible behavior upon stimulation** | **Example Reference(s)** |
| --- | --- | --- | --- | --- |
|  | Active Motor Tasks (*e.g. finger tapping, foot tapping, lip pouting*) | (Sensori)motor cortex  (*Supplementary Motor Area, pyramidal tract*) | Disruption or complete arrest of motor task  Production of involuntary twitching/movement  Production of involuntary sensations over (parts) of the body (*‘tingling’, pain*) | ^1^ |
|  | Language Tasks  (*e.g. word repetition, reading, counting, picture naming, writing, sentence completion, verb generation, spontaneous speech*) | Language-related areas in parietal and frontal lobe  (*Broca, Wernicke, arcuate fasciculus*) | Disruption of speech accuracy or content (different forms of aphasia) or complete speech arrest | ^1–3^ |
|  | Visual Tasks  (*e.g. 8 Hz flickering checkerboard, visuospatial tasks*) | Occipital lobe  (O*ptic radiation*) | Loss of vision (e.g. black spots, spatial neglect) Production of visual content (e.g. ‘seeing stars’) | ^3^ |
|  | Higher Cognitive Tasks  (*calculation, working memory, music, etc.*) | *Debated in literature and dependent on the task, e.g. ­*Calculation: parietal lobe, angular gyrus (*Gerstmann Syndrome*)  Music: temporal lobe, frontal lobe | Disruption or complete arrest of task | ^3,4^ |
| **Anesthetized** | Motor activation through ESM stimulation | Motor cortex  (*Supplementary Motor Area, pyramidal tract*) | Production of involuntary twitching/movements. These need to be detected through visual observation or through EMG (*motor evoked potentials (MEPS)*)  *NB. Most anesthetized functional brain mapping efforts focus on i-fMRI or even EEG/ECoG recordings, often without concomitant ESM. As such, other tasks such as passive motor tasks (through peripheral nerve stimulation)*^5^ *or visual evoked potentials (VEPS) (using flashing lights)*^6^ *can be used for functional mapping.* | ^7^ |

**ESM = Electrocortical Stimulation Mapping, EMG = electromyography, MEPS = motor-evoked potentials, ECoG = Electrocorticography, EEG= Electroencephalography, i-fMRI = intra-operative fMRI, VEPS = visual evoked potentials*

**References**

1. Soloukey S, Vincent AJPE, Satoer DD, et al. Functional Ultrasound (fUS) During Awake Brain Surgery: The Clinical Potential of Intra-Operative Functional and Vascular Brain Mapping. *Front Neurosci*. 2020;13:1384.

2. Collée E, Vincent A, Dirven C, Satoer D. Speech and Language Errors during Awake Brain Surgery and Postoperative Language Outcome in Glioma Patients: A Systematic Review. *Cancer*. 2022;14:5466.

3. Bu L, Lu J, Zhang J, Wu J. Intraoperative Cognitive Mapping Tasks for Direct Electrical Stimulation in Clinical and Neuroscientific Contexts. *Front Hum Neurosci*. 2021;15.

4. Kappen PR, Beshay T, Vincent AJPE, et al. The feasibility and added value of mapping music during awake craniotomy: A systematic review. *Eur J Neurosci*. 2022;55(2).

5. Gasser T, Ganslandt O, Sandalcioglu E, Stolke D, Fahlbusch R, Nimsky C. Intraoperative functional MRI: Implementation and preliminary experience. *Neuroimage*. 2005;26(3):685-693.

6. Gutzwiller EM, Cabrilo I, Radovanovic I, Schaller K, Boëx C. Intraoperative monitoring with visual evoked potentials for brain surgeries. *J Neurosurg*. 2019;130(2).

7. Sanmillan JL, Fernández-Coello A, Fernández-Conejero I, Plans G, Gabarrós A. Functional approach using intraoperative brain mapping and neurophysiological monitoring for the surgical treatment of brain metastases in the central region. *J Neurosurg*. 2017;126(3):698-707.
